# Comprehensive analysis of LASS6 expression and prognostic value in ovarian cancer

**DOI:** 10.1186/s13048-021-00868-z

**Published:** 2021-09-07

**Authors:** Jinshan Xing, Jingyan Yi

**Affiliations:** 1 Department of Neurosurgery, The Affiliated Traditional Chinese Medicine Hospital of Southwest Medical University, Luzhou, Sichuan 646000 China; 2 Department of Medical Cell Biology and Genetics, School of Basic Medical Sciences, Southwest Medical University, Luzhou, Sichuan 646000 China

**Keywords:** LASS6, Ovarian cancer, Prognosis, Bioinformatics, PPI, Calcium ion

## Abstract

**Background:**

Ceramide plays an important role in the occurrence and development of tumor. The synthesis of ceramide needs the participation of LASS. Current studies have shown that different LASS family members play different functions in tumors, especially LASS6, has been proved to play a key role in breast cancer, gastric cancer, melanoma and so on, but the research on ovarian cancer is very limited.

**Methods:**

Bioinformatics web resources, including Oncomine, UALCAN, Kaplan–Meier Plotter and TIMER were used to analyze the expression profile, prognostic value and immune infiltration of LASS6. The related genes of LASS6 in ovarian cancer were mined by Regulome Explorer and LinkedOmics database, and cluster analysis was done by DAVID. The PPI network involving LASS6 was constructed by STRING database. Finally, the correlation between 10 genes and LASS6 was analyzed by GEPIA database, and their prognostic value in ovarian cancer was analyzed by Kaplan–Meier plotter.

**Results:**

The expression of LASS6 was up-regulated in ovarian cancer, which was related to the progression and poor prognosis of ovarian cancer. Through GO/KEGG cluster analysis, we also found that LASS6 may affect calcium ion channel and its transport pathways. The analysis of regulatory network involved in LASS6 showed that the high mRNAs of 7 key genes were associated with poor prognosis of OS in patients with ovarian cancer, among which DEGS1 was the most significant.

**Conclusions:**

LASS6 may play an important role in the regulation of calcium pathway and become a new therapeutic target and potential prognostic marker in ovarian cancer.

**Supplementary Information:**

The online version contains supplementary material available at 10.1186/s13048-021-00868-z.

## Background

Ovarian cancer, endometrial cancer and cervical cancer are the three major gynecological malignant tumors that afflict women. Among them, ovarian cancer has the highest mortality rate, causing more than 150,000 deaths worldwide every year [19, 24, 48]. The five-year average survival rate of ovarian cancer decreases gradually with the increase of clinical stages. Due to the lack of early diagnosis and treatment, 75% of ovarian cancer patients are in stage III and stage IV at the time of diagnosis, and the survival rate is less than 30% [2]. Serous ovarian cancer accounts for the majority of many subtypes of ovarian cancer, and it is divided into high-grade and low-grade ovarian cancer. high-grade serous ovarian cancer has high invasiveness and high mortality, which is the largest subtype of death in patients with ovarian cancer. At present, the standard treatment for patients with ovarian cancer is the combination of platinum and taxane after tumor resection, which usually works well at first, but due to the genetic aberration and high proliferation of ovarian cancer cells, especially high-grade serous ovarian cancer, 70% of patients have drug resistance and recurrence, which is one of the reasons for the low survival rate of ovarian cancer [1, 20, 31]. Because of the limitations of chemotherapeutic drugs in anticancer therapy, targeted therapy has been proposed and gradually applied to various diseases, but the mechanism of targeted drugs is difficult to be observed. Therefore, we urgently need to find reliable biomarkers to analyze the prognosis of clinical treatment and adjust the treatment strategy.

Ceramide synthase (CerS/LASS) is mainly involved in the synthesis of ceramide [4]. Ceramide is the backbone of all sphingolipids and the second messenger of biological activity, which is closely related to cell proliferation, apoptosis, senescence and autophagy [13, 18]. So far, six kinds of LASS proteins have been identified in human [27]. Among them, LASS2, LASS5 and LASS6 have conserved N-glycosylation motifs, which can be modified by ASN-19, ASN-26 and ASN-18, respectively [25]. Each member of the LASS family gives priority to the synthesis of dihydroxyceramides with different fatty acid chain lengths. LASS1/4 mainly synthesizes ceramides containing C18 fatty acid chains, while LASS5/6 prefers to synthesize C16 ceramides and C12/C14 fatty acid chain ceramides [9, 8, 17, 34, 37]. LASS has the function of prolonging life, and ceramide synthesized by LASS is considered to be of great value in tumor therapy, from which it can be inferred that members of the LASS family have potential anticancer ability. Previous studies have shown that LASS family plays a key role in breast cancer, head and neck squamous cell carcinoma, glioma and so on, but the research on ovarian cancer is very limited [10, 15, 26, 32, 36, 38]. Through data mining, we found that only LASS6 was significantly differentially expressed in ovarian cancer. However, due to the late discovery of LASS6, there is still a lack of important information about its mechanism, and a lot of space for study. It is precisely because the expression of LASS6 is cancer-specific, we speculate that it may play an important regulatory role in ovarian cancer, which is worth exploring and digging.

LASS6 has been studied in other cancers. Bao et al. found that LASS6-AS1 can enhance the stability of LASS6 mRNA by binding to IGF2BP3, make it highly expressed in breast tumor cells and tissues, and play a role in promoting proliferation and inhibiting apoptosis. It acts as a malignant promoter [3]. Similarly, there is a high expression of LASS6, in gastric cancer, which can regulate cell cycle control and metastasis-related proteins through SOCS2/JAK2/STAT3 signaling pathway to affect cell dryness, thus promoting cell proliferation and diffusion and the growth of xenografts. It can be used as a potential biomarker for prognostic analysis of patients with gastric cancer [49]. On the other hand, LASS6 seems to function as a tumor suppressor gene. Some studies have shown that LASS6 is a direct transcriptional target of p53 and participates in p53-dependent cellular stress response [11]. In melanoma, LASS6 has been found to up-regulate the activity of glycolysis-related enzymes and the expression of glycolysis-related genes (including GLUT1 and MCT1), and inhibit cell proliferation and invasion [44]. Breast cancer studies have shown that LASS6 reduces the level of phosphorylation of Akt/mTOR/ERK in cells and negatively regulates tumor-promoting factors SphK1 and S1PR2 [16]. Similarly, knockout of LASS6 blocked Caspase cascade apoptosis, down-regulation of focal adhesion kinase and subsequent secondary necrosis, such as FAK deletion and plasma membrane rupture, induced by tumor necrosis factor TNFα [14]. In colon cancer, knocking down LASS6 leads to a specific decrease in C16 neuramides, which protects tumor cells from TRAIL-mediated apoptosis and interferes with the entry of active Caspase-3 into the nucleus [52].

Some other studies have also proved the biological function of LASS. LASS6 can mediate the transcriptional activation of acid ceramidase in colon cancer in a JNK-dependent manner [47]. In gastric cancer, vorinostat alone can promote the acetylation of LASS6 in cells. After treatment with sorafenib, the synthesis of LASS6 increases and the expression of CD95 in tumor cells is activated. This process involves a signal mechanism dependent on reactive oxygen species [30]. A new study shows that C16 ceramide produced by LASS6 can accumulate in mitochondria and mitochondrial-related membranes and inhibit mitochondrial β-oxidation in the liver and brown adipose tissue, while LASS6-deficient mice can avoid obesity and glucose tolerance caused by high-fat foods [12].

In this study, we will use bioinformatics sites such as UALCAN, Oncomine, Kaplan–Meier Plotter, Human Protein Atlas (HPA) and other studies to study the expression of LASS6 in different clinicopathological parameters of ovarian cancer and the correlation with the level of immune infiltration, and to explore the prognostic value of LASS6. In addition, we will also mine the genes related to the expression of LASS6 in ovarian cancer, through GO/KEGG cluster analysis, count the possible pathways and life activities affected by LASS6, and speculate its possible mechanism and pathway. Finally, we build the protein–protein interaction (PPI) network of LASS6 through STRING, which provides further theoretical basis and potential direction for further exploration of the mechanism.

## Materials and methods

### Oncomine analysis

Oncomine (https://www.oncomine.org/) is a powerful bioinformatics platform with the largest tumor gene chip database and online data mining functions in the world, including 715 data sets and 86,733 samples. It aims to collect, standardize, analyze and transmit the expression data of normal and cancer samples to the biomedical research community [33]. We used Oncomine database to obtain the mRNA level of LASS gene family in different types of cancer, and analyzed the expression of LASS6 in different ovarian cancer databases.

### Kaplan–Meier plotter analysis

Kaplan–Meier Plotter database (http://kmplot.com/analysis/) can evaluate the effect of 54,000 genes on the survival rates of 21 cancer types, including breast cancer (*n* = 6234), ovarian cancer (*n* = 2190), lung cancer (*n* = 3452) and gastric cancer (*n* = 1440). It is an online tool commonly used to draw survival curves [28]. The prognostic value of LASS gene family in ovarian cancer is estimated by Kaplan–Meier Plotter. Specifically, the genes to be tested are inputted into the database and divided into high and low groups according to the expression level of genes to be tested in the tumor, and then automatically generate Kaplan–Meier survival map. We are calculating the overall survival (OS).

### UALCAN analysis

UALCAN (http://ualcan.path.uab.edu/) is an open portal that can analyze and mine TCGA cancer and CPTAC clinical protein data online, and compare gene expression between normal samples and tumors in different tumor subsets [5]. We used its "expression analysis" module and "Ovarian cancer" data set to detect the prognostic value of LASS6. We will analyze the differential expression of LASS6 in terms of sample type, cancer stage, patient age and tumor grade.

### HPA analysis

HPA (https://www.proteinatlas.org/) is a biological research platform based on TCGA database, which uses a variety of combinatorial techniques to characterize the expression of proteins in tissues and cells, including the localization and distribution of thousands of proteins in various cancer tissues [46]. In this study, we will use HPA database to analyze the immunohistochemical differential expression of LASS6 in tumor tissues and normal tissues, the antibody number is HPA044683.

### Tumor Immune Estimation Resource (TIMER) analysis

TIMER (https://cistrome.shinyapps.io/timer/) database is mainly used to systematically analyze immune infiltration of different cancer types. It includes 10,897 samples from 32 cancer types. Through RNA-seq expression profile data, we can accurately quantify tumor purity and immune infiltration level, and evaluate the correlation between invasion and clinical prognosis [21, 22]. In this study, we explored the relationship between LASS6 and purity, B cell, CD8 + T cell, CD4 + T cell, macrophage, neutrophil and dendritic cell immune cells in ovarian cancer. Log2 TPM was used to show the level of gene expression.

### Regulome analysis

Regulome Explorer (http://explorer.cancerregulome.org/) is a global cancer analysis tool in TCGA database, which can draw a network map to show the relationship between target gene and tumor genome according to the correlation among gene, DNA methylation, somatic copy number, somatic mutation and protein level. We will use this tool to map the gene interaction network of LASS6 in ovarian cancer. The pairwise correlation between the two genes was analyzed by Spearman correlation, and only the *P* > -log10 genes appeared in the network map.

### LinkedOmics analysis

LinkedOmics database (http://www.linkedomics.org/login.php) contains 11,158 cases of TCGA, 32 kinds of cancer multi-group data and clinical data, can analyze and compare cancer multi-group data within and across tumor types [50]. We used the Link Finder module in LinkedOmics to screen the differentially expressed genes related to LASS6 in ovarian cancer from the TCGA_OV dataset.

### Database for Annotation, Visualization, and Integrated Discovery (DAVID) analysis

DAVID (https://david.ncifcrf.gov/home.jsp) is a database of bioinformatics tools, which can annotate gene sets, analyze them statistically, and enrich the most significant biological information [6]. In this study, we were able to carry out GO and KEGG cluster analysis of the genes related to LASS6 obtained by LinkedOmics through DAVID. GO analysis included three parts: molecular function (MF), biological process (BP) and cell composition (CC), and the top five pathways of each part were statistically analyzed according to P value. The GO/KEGG cluster analysis diagram was drawn by GraphPad Prism software.

### PPI network analysis

STRING (https://string-db.org/) is an interactive and visual online prediction tool, which is mainly used to explore the interaction between proteins [43]. The PPI analysis of LASS6 developed by STRING database is provided.

### Gene Expression Profiling Interaction Analysis (GEPIA)

GEPIA (http://gepia.cancer-pku.cn/) is an online analysis website based on transcriptome sequencing data from 9736 tumor samples and 8587 normal samples from TCGA and GTEx databases, which can be used to evaluate the correlation between the two genes in cancer [45]. In this study, we obtained the co-expression of LASS6 and 10 genes encoding PPI network protein in ovarian cancer through GEPIA database.

### Statistical analysis

Student’s test was used to analyze the gene expression data of Oncomine and UALCAN databases. Spearman correlation analysis was used to evaluate TIMER and Regulome databases. Pearson’s correlation test was used to count LinkedOmics and GEPIA databases. Survival curves were drawn by Kaplan–Meier curves and compared with log-rank test. *P* < 0.05 is considered to be statistically significant.

## Results

### The expression of LASS family genes in cancer

**Fig. 1:**
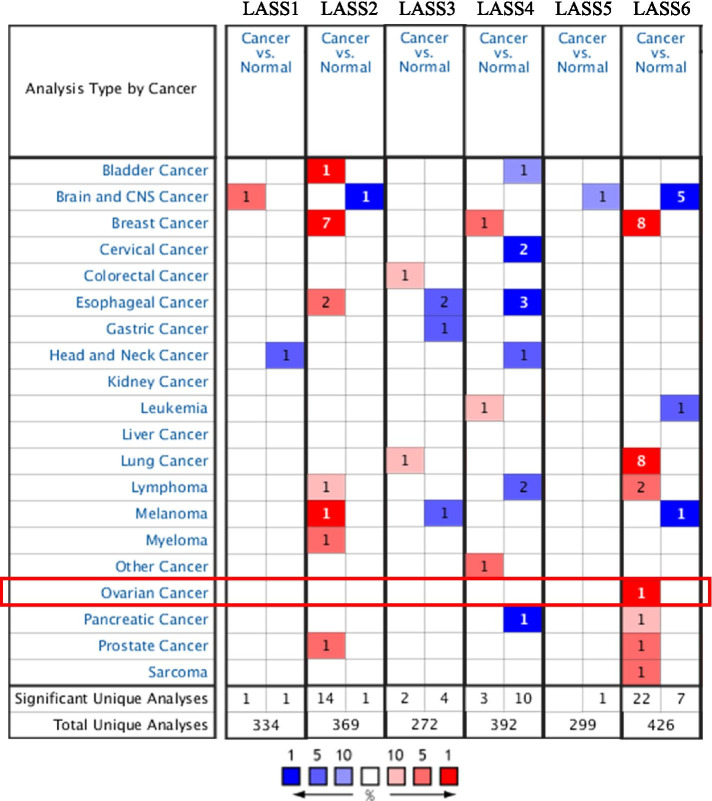
Expression of LASS family genes in different types of cancers. Red and blue represent significantly up-regulated and down-regulated mRNA levels of LASS family genes, respectively

In order to study the difference of LASS family gene expression between tumor samples and normal tissues, we used Oncomine database to analyze the mRNA levels of 6 LASS family genes in different tumor types (Fig. 1). The results showed that the expression of LASS gene was different in all types of tumors and their corresponding normal tissues. In breast cancer, LASS2, LASS4 and LASS6 are highly expressed. In lung cancer, LASS3 and LASS6 are highly expressed. In esophageal carcinoma, the expression of LASS2 is high, while the expression of LASS3 and LASS4 is down-regulated. In lymphoid carcinoma, the expression of LASS2 and LASS6 is high, while the expression of LASS4 is low. Interestingly, compared with other LASS family genes, LASS6 has a higher frequency of abnormal expression in cancer, and only its transcriptional expression is significantly up-regulated in ovarian cancer. The database includes 334, 369, 272, 392, 299 and 426 unique analyses of LASS1, LASS2, LASS3, LASS4, LASS5 and LASS6, respectively.

### Survival analysis of LASS family genes in ovarian cancer

**Fig. 2:**
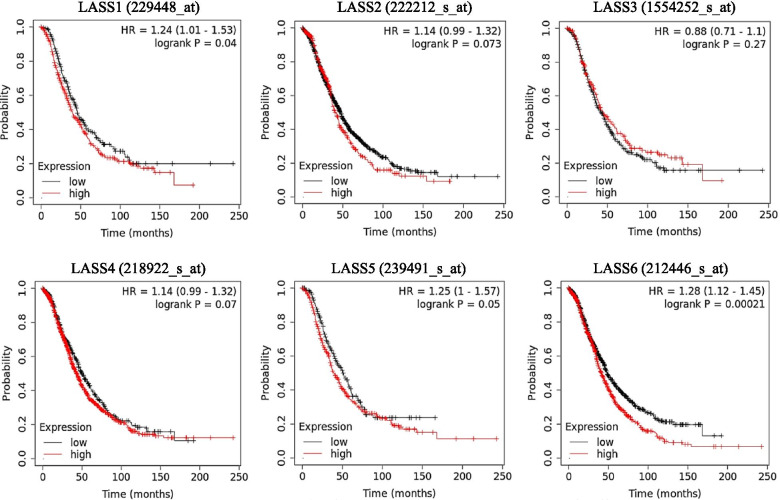
Survival Analysis of LASS family genes in ovarian cancer. The prognostic values (OS) of the LASS family genes in ovarian cancer was determined by Kaplan–Meier plotter

In order to study whether the expression of LASS family genes affects the survival of ovarian cancer patients, we used the Kaplan–Meier Plotter database to compare the OS of 655 ovarian cancer patients with high or low expression of six LASS family genes in the TCGA dataset (Fig. 2). The results showed that the expression of LASS1, LASS5 and LASS6 had an effect on the OS and prognosis of patients with ovarian cancer. It is worth noting that the high expression of LASS6 (*P* = 0.00021) can significantly affect the prognosis of ovarian cancer compared with LASS1 (*P* = 0.04) and LASS5 (*P* = 0.05). There was no significant correlation between the expression of LASS2, LASS3 and LASS4 and the prognosis of ovarian cancer. These findings suggest that the expression of LASS6 may be a prognostic indicator of the risk of death in patients with ovarian cancer and its relationship with the prognosis value of ovarian cancer is worth exploring.

### The expression of LASS6 in ovarian cancer

**Fig. 3:**
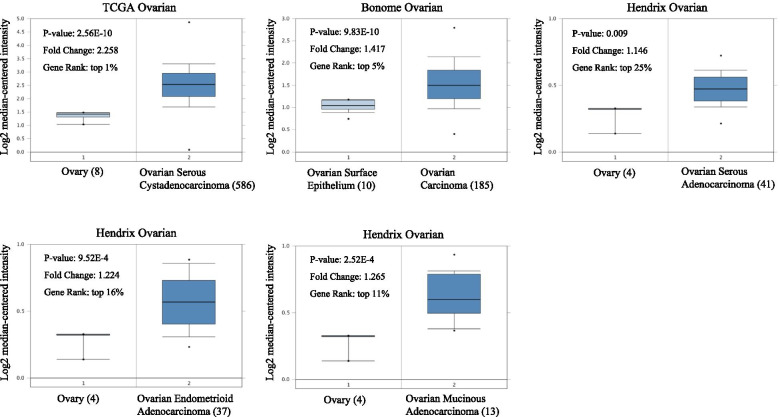
Expression of LASS6 in ovarian cancer. The box plot showing fold changes and related *P* values of LASS6 levels in normal ovary and ovarian cancer based on Oncomine, respectively in the TCGA, Bonome and Hendrix databases

**Fig. 4:**
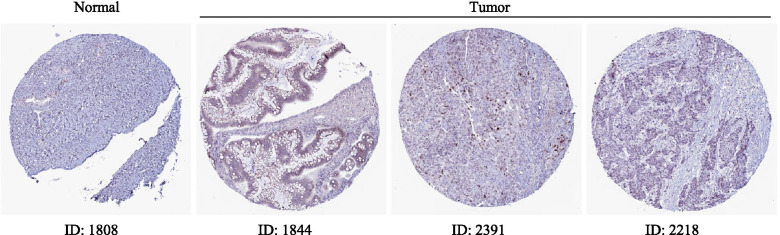
In situ expression of LASS6 in ovarian cancer. Using the immunohistochemical data provided by HPA database, the expression of LASS6 in normal ovarian tissue and ovarian cancer was analyzed at protein level

We analyzed the transcriptional level of LASS6 in ovarian cancer and normal tissues from a series of ovarian tumor databases (Fig. 3). The results showed that in the three TCGA, Bonome and Hendrix ovarian cancer databases, the expression of LASS6 in tumor tissues was significantly higher than that in normal ovarian tissues. Among them, the Hendrix database included serous, endometrioid and mucinous subsets, which were statistically significant, and *P* values were 0.009, 9.52E-4 and 2.52E-4, respectively. In order to understand the expression of LASS6 protein in ovarian cancer tissues, we used HPA database to study the difference of LASS6 expression between human ovarian cancer and normal ovarian tissues (Fig. 4). The antibodies used for staining in both groups were HPA044683. We found that the staining intensity of LASS6 in tumor tissues was significantly higher than that in normal tissues, suggesting the high expression of LASS6 in ovarian cancer tissues.

### Expression of LASS6 in human ovarian cancer subtypes

**Fig. 5:**
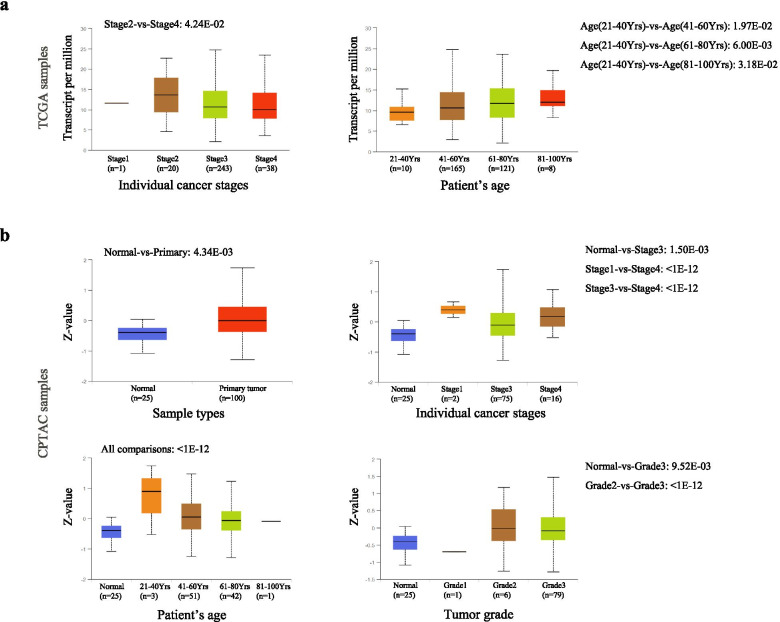
Differential expression of LASS6 in human ovarian cancer subtypes. **A** Based on TCGA database, the differences of LASS6 transcription levels in different clinical stages and ages of patients with ovarian cancer were shown. **B** Based on the CPTAC database, the differences of LASS6 protein levels among subtypes of ovarian cancer patients classified by sample type, clinical stage, patient age, and tumor grade were shown

In order to further demonstrate the specificity of LASS6 in ovarian cancer, we integrated various clinical factors of ovarian cancer samples, such as clinical stage and patient age, in the TCGA database, and compared the differences in LASS6 transcription levels among different groups (Fig. 5A). The results showed that according to the clinical stage, the transcription level of LASS6 in stage 2–4 was higher than that in normal tissues, and the expression was the highest in stage 2. The statistical significance of stage 2 vs stage 4 was found (*P* = 4.24E-02). According to the age of the patients, it was found that the transcriptional level of LASS6 increased with age. The statistical significance of 21–40 vs 41–60 years old, 21–40 vs 61–80 years old and 21–40 vs 81–100 years old were significant (*P* = 1.97E-02, *P* = 6.00E-0, *P* = 3.18E-02, respectively).

At the same time, we compared the protein expression levels of LASS6 in different subtypes of ovarian cancer patients in the CPTAC database (Fig. 5B). The results showed that according to the sample type, compared with normal ovarian tissue, LASS6 showed significantly higher expression in primary tumor tissues, and the statistical significance of normal vs primary tumor was significant (*P* = 4.34E-03). According to the clinical stage, the protein level of LASS6 was higher than that of normal tissue in stage 1, 3 and 4, and the expression was the highest in stage 1. The statistical significance of normal vs stage 3, stage 1 vs stage 4 and stage 3 vs stage 4 were significant (*P* = 1.50 E-03, *P* < 1E-12, *P* < 1E-12, respectively). According to the age of the patients, we found that the expression of LASS6 was the highest in people aged 21–40 years old, and the pairwise comparison *P* value of all groups was less than 1E-12. According to the tumor grade, the protein level of LASS6 in grade 2–3 tumors was significantly higher than that in normal tissues. The statistical analysis of normal vs grade 3 and grade 2 vs grade 3 were *P* = 9.52 E-03 and *P* < 1E-12.

### Correlation between LASS6 expression and immune infiltration in ovarian cancer

**Fig. 6:**
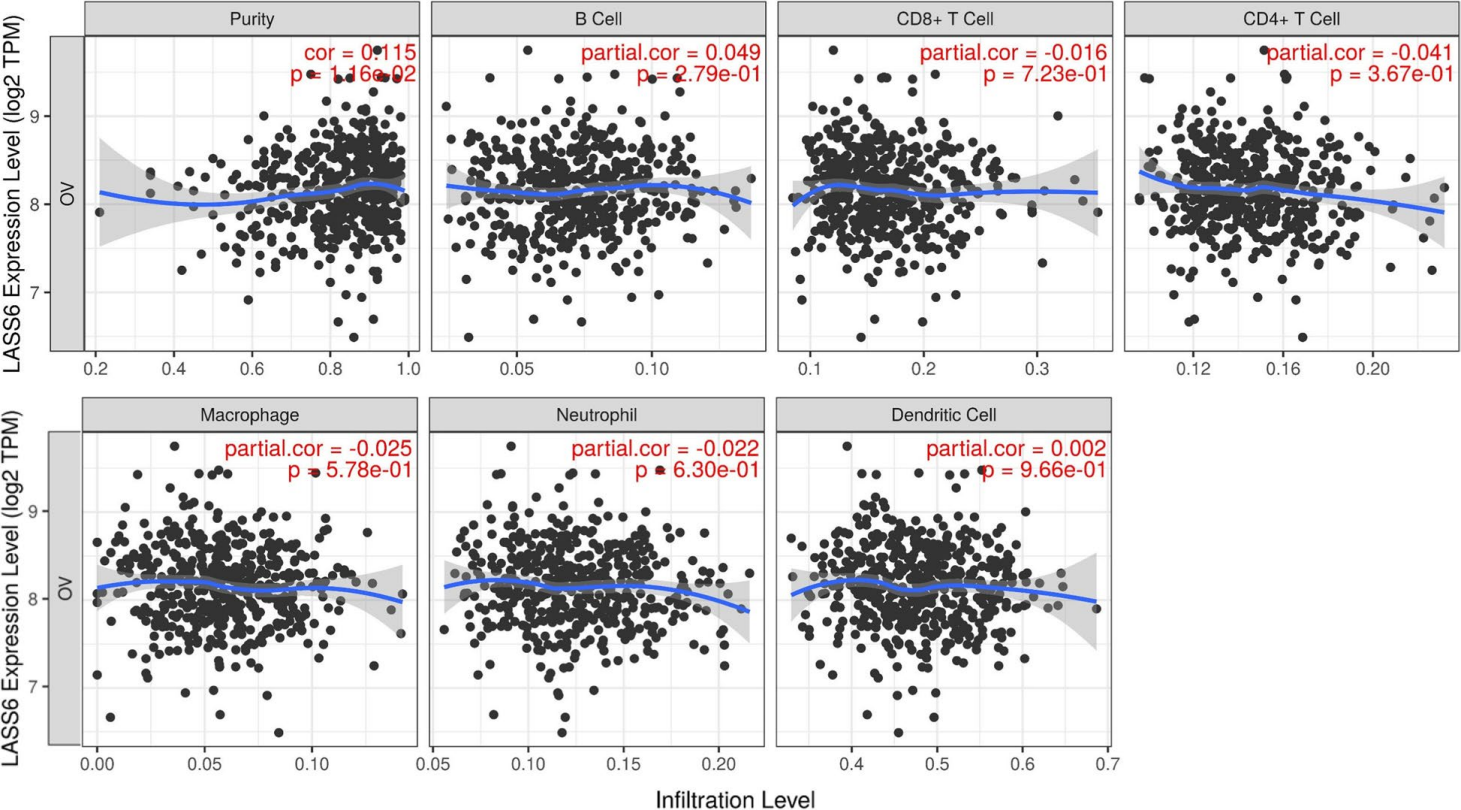
Correlation between LASS6 expression and immune infiltration in ovarian cancer. The relationship between LASS6 expression and purity, B cells, CD8 + T cells, CD4 + T cells, macrophages, neutrophils and dendritic cells were detected

Tumor infiltrating lymphocytes have been used to predict the status and survival of sentinel lymph nodes in cancer. Therefore, we used the TIMER database to study the relationship between LASS6 expression and immune infiltration in patients with ovarian cancer (Fig. 6). The results showed that the expression level of LASS6 in ovarian cancer was only positively correlated with tumor Purity (*r* = 0.115, *P* = 1.16E-02).

### The correlation between LASS6 and other genes in ovarian cancer

**Fig. 7:**
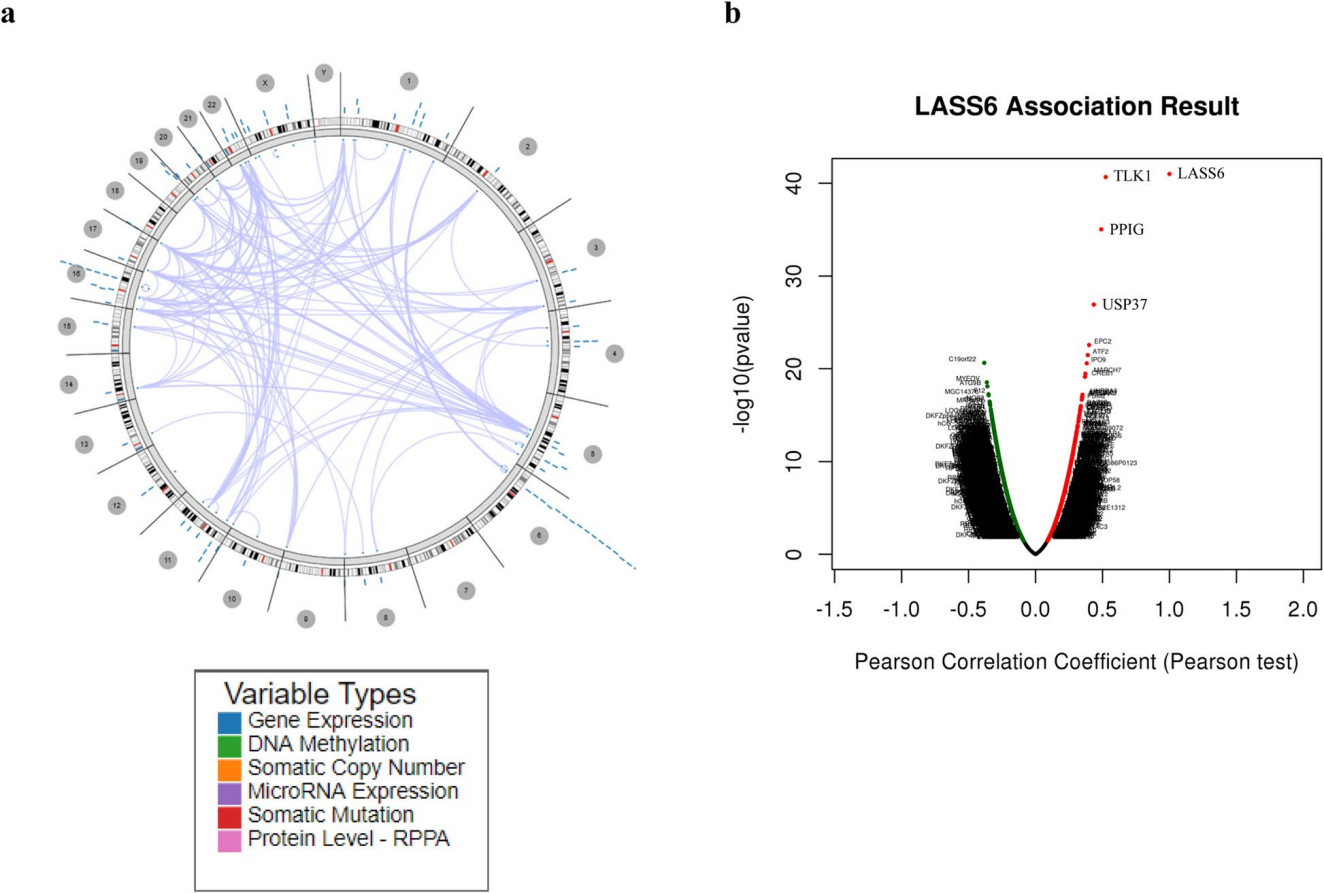
Correlation between LASS6 and other genes in ovarian cancer. **A** The circus plot displayed the expression of LASS6 and its correlation with other genes in ovarian cancer, which is mapped according to the association between gene, DNA methylation, somatic copy number, somatic mutation and protein level. **B** The volcano map showed the correlations between LASS6 and genes differentially expressed in ovarian cancer, red indicates positively correlated genes and green indicates negatively correlated genes

Using Regulome Explorer, we further analyzed the genomic location of human ovarian cancer and the correlation between some genes and LASS6. Based on the association between gene, DNA methylation, somatic copy number, somatic mutation and protein level, a circus map was drawn to show the network of interaction between LASS6 and other genes, showing *P* > -log10 genes (Fig. 7A). TCGA data show that there is a significant correlation between many genes detected in ovarian cancer and LASS6, indicating that LASS6 is closely related to the genome of ovarian cancer.

In order to further study the potential role of differentially expressed LASS6 in ovarian cancer, we used the Link Finder module of LinkedOmics to analyze the RNA-seq data of 569 patients with TCGA, and the results were analyzed by Pearson test. In the volcanic map, dark red dots represent genes that are significantly positively correlated with LASS6, while dark green dots represent genes that are significantly negatively correlated with LASS6 (FDR < 0.01) (Fig. 7B). We found that there was a significant positive correlation between 3 genes (TLK1, PPIG and USP37) and LASS6. The first 10 genes with positive correlation and negative correlation are shown in Additional file 1.

### GO and KEGG analysis of LASS6 related genes in ovarian cancer

DAVID was used to annotate the gene sets associated with LASS6 in ovarian cancer, and GO and KEGG cluster analysis were done. GO analysis included three groups: BP, MF and CC. The obtained results select the top five pathways or life activities of each type according to the P value, and draw a broken line histogram with GraphPad Prism (Additional file 2, Fig. 8). The results showed that BP group analyzed by GO enriched calcium ion transmembrane transport, membrane potential regulation, social behavior, ion transmembrane transport regulation and spinal cord related neuronal differentiation, while MF group enriched RNA polymerase II distal enhancer sequence specific DNA binding, serine peptidase activity, eye lens structure, microtubule binding and ATP-dependent microtubule movement. In CC group, synaptic vesicle membrane, kinesin complex, presynaptic membrane and keratin filament were enriched, while KEGG was enriched by neuroactive ligand-receptor interaction, calcium signal pathway, serotonin-synapse, cAMP signal pathway and cell cycle.

**Fig. 8:**
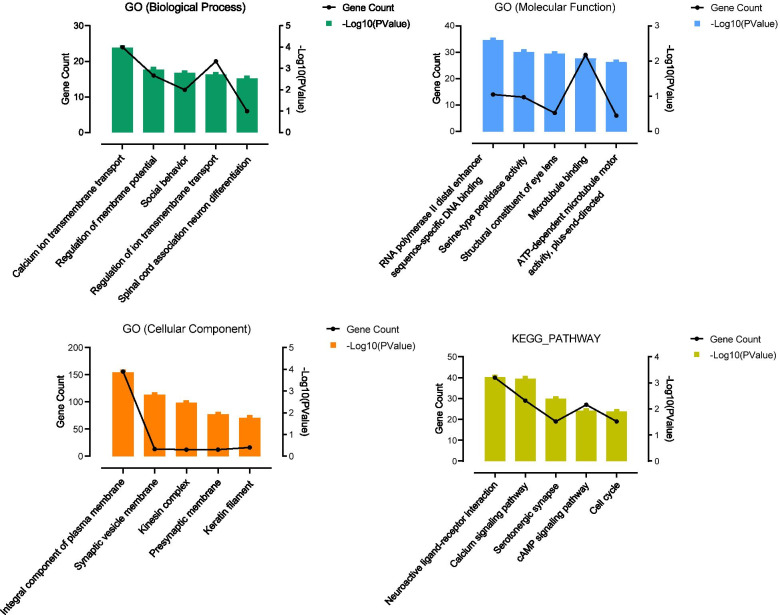
GO/KEGG analysis of LASS6 related genes in ovarian cancer. DAVID analyzed the GO annotation and KEGG pathway in which LASS6 coexpression genes were significantly enriched in ovarian cancer. GO annotation included three groups: BP, MF and CC. The obtained results select the top five pathways or life activities of each type according to the *P* value, and draw a broken line histogram with GraphPad Prism

### The construction of PPI network involved in LASS6

**Fig. 9:**
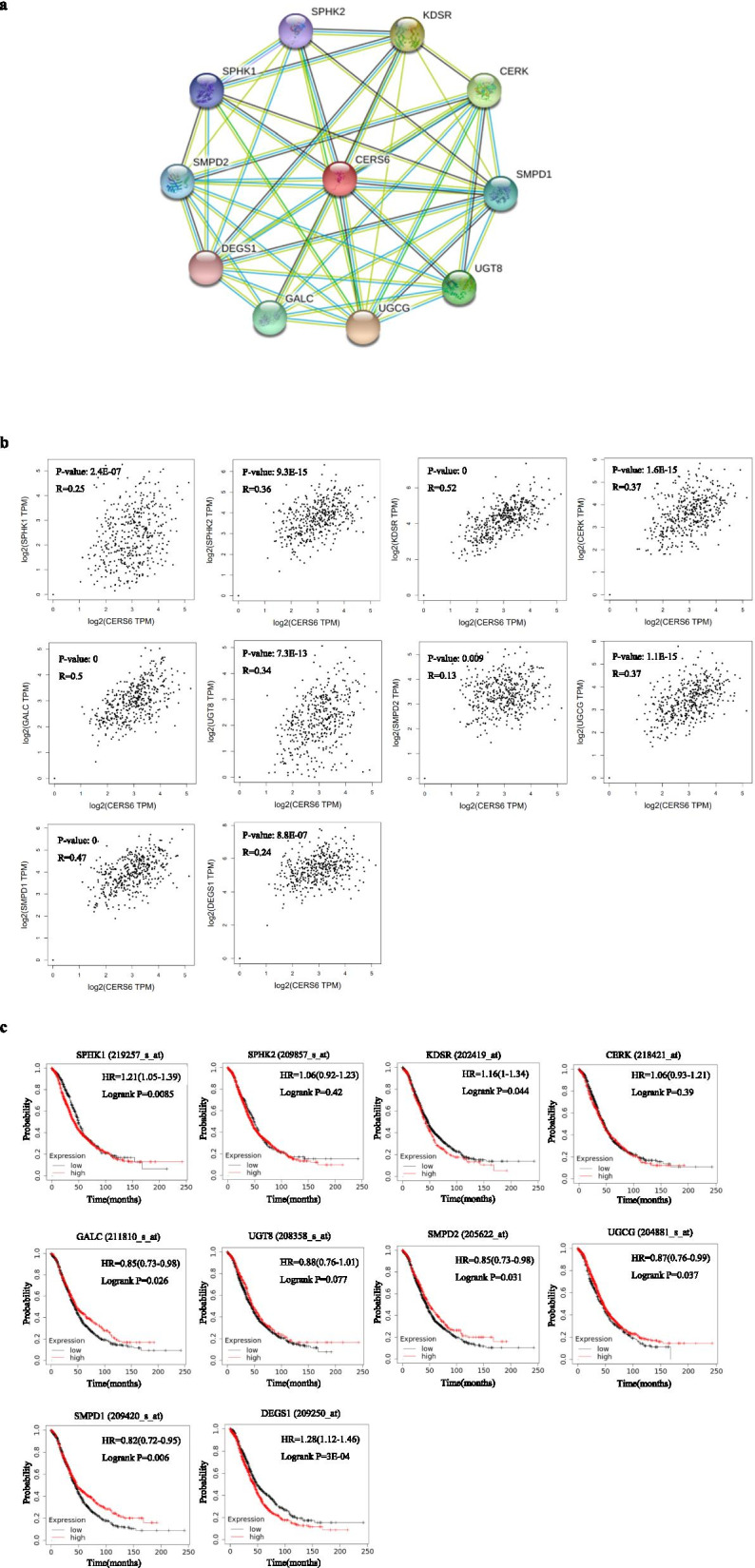
Expression correlation and survival analysis of LASS6 with genes in the LASS6-involved PPI network. **A** The PPI networks of LASS6 constructed using STRING database. **B** The expression relationship between 10 correlated genes and LASS6 in ovarian cancer assessed by GEPIA database. **C** The prognostic values (OS) of the 10 LASS6-related genes in ovarian cancer was determined by Kaplan–Meier plotter

In order to dig into the molecular mechanism, we used STRING database to build a PPI network involving LASS6. Based on the degree score, the top 10 proteins were SPHK1, SPHK2, KDSR, SMPD1, SMPD2, DEGS1, UGCG, CERK, GALC and UGT8, which were identified as potential research priorities (Fig. 9A). In addition, we also used GEPIA database to evaluate the correlation between genes encoding proteins in PPI networks and LASS6 mRNA expression (Fig. 9B). The results showed that the expression of LASS6 was positively correlated with the expression of all 10 genes in ovarian cancer. In order to evaluate the prognostic value, we compared the OS of 10 genes in ovarian cancer using Kaplan–Meier Plotter database (Fig. 9C). Survival analysis showed that the high levels of SPHK1, KDSR, SMPD1, GALC, SMPD2, UGCG and DEGS1 were associated with poor prognosis of OS in patients with ovarian cancer, among which DEGS1 was the most significant (*P* = 3E-04). However, there was no significant difference in OS between SPHK2, CERK and UGT8 in ovarian cancer.

## Discussion

LASS6 is a member of the ceramide synthase family and participates in the synthesis of ceramide. Many studies have shown that LASS6 plays an important role in a variety of cancers. Due to the emergence of heterogeneity, LASS6 may have both carcinogenic and anticancer effects. However, as far as we know, there is still a lack of research on the expression, function and mechanism of LASS6 in ovarian cancer. Therefore, this study was carried out.

In this study, we used Oncomine database to analyze the expression of 6 LASS family genes in cancer and found that only LASS6 was significantly overexpressed in ovarian cancer. Subsequently, we used Kaplan–Meier Plotter database to evaluate the prognostic value of LASS family genes in ovarian cancer, and the results were consistent with Oncomine database, only the high expression of LASS6 was significantly negatively correlated with OS. We also compared the mRNA expression level of LASS6 in tumor tissues and normal ovarian tissues in TCGA, Bonome and Hendrix ovarian cancer databases. The results showed that LASS6 was significantly overexpressed in tumor tissues. Some studies have shown that LASS6 is extremely active in breast cancer and gastric cancer, and plays a role in promoting proliferation and inhibiting apoptosis, so it belongs to malignant promoter [3, 49]. We studied the difference of LASS6 protein expression between ovarian cancer and normal ovarian tissues by HPA database. We found that the staining intensity of LASS6 in tumor tissues was significantly higher than that in normal tissues, suggesting that the high expression of LASS6 in ovarian cancer tissues partly supported the accuracy of our previous bioinformatics analysis. All these findings suggest that LASS6 may be an important oncogene in ovarian cancer, and a potential biomarker for the diagnosis of ovarian cancer patients. Therefore, LASS6 may become a promising therapeutic target in the field of ovarian cancer treatment in the future.

Subsequently, we based on partial clinicopathological features, including sample type, clinical stage, patient age and tumor grade in the expression of LASS6. Generally speaking, LASS6 is highly expressed in the early stage of ovarian cancer, which is related to the occurrence and development of ovarian cancer. In this study, we also used TIMER database to analyze the relationship between LASS6 and immune infiltration. Interestingly, the expression level of LASS6 in ovarian cancer was only positively correlated with tumor purity, and no correlation was found with other immune cells, suggesting that LASS6 may not be affected by the immune microenvironment of ovarian cancer. Genes usually interact with other genes to play their biological role. In order to understand the mechanism of LASS6, we used Regulome Explorer and LinkedOmics database to mine the gene set associated with LASS6 in ovarian cancer. The results showed that there was a close relationship between LASS6 and ovarian cancer genome. Additional file 1 showed that there was a significant positive and negative correlation gene set. We found that TLK1, PPIG and USP37 had the highest correlation coefficient, and the three genes were all positive correlation genes. Through the DAVID, we got the GO/KEGG cluster analysis data, the results showed that there are two clearly related to calcium, which are the calcium transport across the membrane in GO analysis and the calcium signal pathway in KEGG. In addition, the regulation of membrane potential, the regulation of ion transmembrane transport, the components of microtubule binding, kinesin complex and plasma membrane in GO analysis, as well as the cell cycle of KEGG analysis all suggest that the physiological role of LASS6 in ovarian cancer may be related to calcium-related transport process, which is dynamic. Calcium signaling is a key regulator of many cellular processes, which has been proved to be related to cancer progression and plays a key role in many cancers, mainly invasion pathway and cancer cell migration [35, 40]. Moreover, the induction of FOS, JUN and MYC by calcium ions in cell cycle G 1 has been found for a long time [35].

In order to understand the molecular mechanism of LASS6, we use STRING database to build a PPI network involving LASS6. The 10 proteins with the highest scores were SPHK1, SPHK2, KDSR, SMPD1, SMPD2, DEGS1, UGCG, CERK, GALC and UGT8, respectively. Correlation analysis showed that LASS6 was significantly positively correlated with 10 genes involved in the regulatory network, which was identified as a potential research focus and may play a key role in our study of the mechanism of LASS6. In particular, the high expression of DEGS1, is significantly related to the poor prognosis (OS) of patients with ovarian cancer. DEGS1, whose full name is Delta 4-Desaturase, Sphingolipid 1, is a member of the family of fatty acid desaturases that encode membrane fatty acids [42, 41]. It is responsible for inserting double bonds into specific sites of fatty acids, which may be located in endoplasmic reticulum, mitochondrial membrane, and participate in many biological processes, such as lipid metabolism, sphingolipid metabolism and leukocyte immunity [7, 29, 53].

In terms of drug resistance, studies on T-cell acute lymphatic cancer have shown that LASS6 can bind to CD95/Fas and alter the toxicity of pan-Bcl-2 inhibitor ABT-737 to cells through the external pathway of apoptosis [51]. Similarly, Senkal et al. found that C16 ceramide produced by LASS6 is a survival factor that protects head and neck squamous cell carcinoma cells from apoptosis induced by endoplasmic reticulum stress by selectively regulating the ATF6 / CHOP axis and inducing the growth of xenografted tumors in mice [39]. Interestingly, Sheng et al. found that overexpression of LASS6 enhanced mitochondrial fission and apoptosis in cisplatin-resistant oral squamous cells and attenuated cisplatin-induced autophagy [23]. In short, our study provides preliminary data for further study of the function and related mechanism of LASS6, in order to explore its diagnostic and prognostic significance as a therapeutic target and clinical biomarker.

## Conclusions

To sum up, we comprehensively analyzed the expression and prognostic value of LASS6 in human ovarian cancer. The high expression of LASS6 indicates a poor prognosis of ovarian cancer. By mining the genes related to the expression of LASS6 in ovarian cancer and doing cluster analysis, we found that LASS6 may affect the calcium ion channel and its transport pathway. DEGS1, which is the most closely related PPI regulatory network of LASS6, also provides important clues for LASS6 research. Of course, these hypotheses need to be verified by further experiments. At present, there are few studies on LASS6 in ovarian cancer. Our work can lay a foundation for exploring the biological function of LASS6 in tumor and its effect on clinical drug resistance in the future. At the same time, it is also hopeful to provide potential prognostic targets and new ideas for clinical treatment of ovarian cancer.

## Supplementary Information

**Additional file 1: Table 1.** Positively correlated significant genes. **Table 2.** Negatively correlated significant genes

**Additional file 2: Table 3.**The top 5 GO items related to proteins involved in LASS6 network. **Table 4.** The top 5 KEGG pathways related to proteins involved in LASS6 network

## Data Availability

The data that support the findings of this study are available online. Contact with correspondence author for any data and material on reasonable request.

## Abbreviations

PPI: Protein–protein interaction
HPA: Human Protein Atlas
CerS/LASS: Ceramide synthase
TIMER: Tumor Immune Estimation Resource
DAVID: Database for Annotation, Visualization, and Integrated Discovery
MF: Molecular function
BP: Biological process
CC: Cell composition
GEPIA: Gene Expression Profiling Interaction Analysis
OS: Overall survival
