# Supplementary material for: Comprehensive analysis of LASS6 expression and prognostic value in ovarian cancer
Source: J Ovarian Res. 2021 Sep 7;14:117. doi: 10.1186/s13048-021-00868-z (PMC8422657; doi:10.1186/s13048-021-00868-z)
Supplement: Supplementary file 1 — Additional file 1: Table 1. Positively correlated significant genes. Table 2. Negatively correlated significant genes [file 13048_2021_868_MOESM1_ESM.pdf]

Additional file 1

Table 1 Positively correlated significant genes

Query	Statistic	P-value	FDR (BH)
TLK1	0.523699	2.16E-41	6.95E-38
PPIG	0.490218	9.82E-36	2.10E-32
USP37	0.434721	1.24E-27	1.99E-24
EPC2	0.400202	2.68E-23	3.45E-20
ATF2	0.390739	3.39E-22	3.64E-19
IPO9	0.382929	2.60E-21	2.09E-18
CREB1	0.369489	7.58E-20	4.87E-17
HNRPA3	0.350936	6.21E-18	2.85E-15
NFE2L2	0.349398	8.83E-18	3.35E-15
UGCGL1	0.349379	8.87E-18	3.35E-15

Table 2 Negatively correlated significant genes

Query	Statistic	P-value	FDR (BH)
C19orf22	-0.3834	2.30E-21	2.09E-18
MYEOV	-0.36413	2.79E-19	1.63E-16
ATG9B	-0.35984	7.77E-19	4.16E-16
F12	-0.35149	5.47E-18	2.70E-15
MGC14376	-0.35038	7.05E-18	3.02E-15
C6orf1	-0.34175	4.95E-17	1.45E-14
MRPL54	-0.34072	6.21E-17	1.74E-14
PLLP	-0.33887	9.35E-17	2.50E-14
ITGAE	-0.33458	2.38E-16	5.11E-14
LCTL	-0.33348	3.03E-16	6.03E-14
