# Supplementary material for: Comprehensive analysis of LASS6 expression and prognostic value in ovarian cancer
Source: J Ovarian Res. 2021 Sep 7;14:117. doi: 10.1186/s13048-021-00868-z (PMC8422657; doi:10.1186/s13048-021-00868-z)
Supplement: Supplementary file 2 — Additional file 2: Table 3.The top 5 GO items related to proteins involved in LASS6 network. Table 4. The top 5 KEGG pathways related to proteins involved in LASS6 network [file 13048_2021_868_MOESM2_ESM.pdf]

Additional file 2

Table 3 The top 5 GO items related to proteins involved in LASS6 network

GO-term	Description	P Value
Biological Process (BP)		
GO:0070588	Calcium ion transmembrane transport	1.08E-04
GO:0042391	Regulation of membrane potential	0.001132
GO:0035176	Social behavior	0.001607
GO:0034765	Regulation of ion transmembrane transport	0.001915
GO:0021527	Spinal cord association neuron differentiation	0.002877
Molecular Function (MF)		
GO:0000980	RNA polymerase II distal enhancer sequence-specific DNA binding	0.002526
GO:0008236	Serine-type peptidase activity	0.005509
GO:0005212	Structural constituent of eye lens	0.00618
GO:0008017	Microtubule binding	0.008401
GO:0008574	ATP-dependent microtubule motor activity, plus-end-directed	0.010786
Cell Composition (CC)		
GO:0005887	Integral component of plasma membrane	1.37E-04
GO:0030672	Synaptic vesicle membrane	0.001503
GO:0005871	Kinesin complex	0.003534
GO:0042734	Presynaptic membrane	0.011927
GO:0045095	Keratin filament	0.017128

Table 4 The top 5 KEGG pathways related to proteins involved in LASS6 network

Pathway-term	Description	P Value
KEGG Pathways		
hsa04080	Neuroactive ligand-receptor interaction	6.04E-04
hsa04020	Calcium signaling pathway	6.86E-04
hsa04726	Serotonergic synapse	0.004033
hsa04024	cAMP signaling pathway	0.011664
hsa04110	Cell cycle	0.01275
